# Exposure to Multicultural Context Affects Neural Response to Out-Group Faces: A Functional Magnetic Resonance Imaging Study

**DOI:** 10.3390/s23084030

**Published:** 2023-04-16

**Authors:** Alessandro Carollo, Paola Rigo, Andrea Bizzego, Albert Lee, Peipei Setoh, Gianluca Esposito

**Affiliations:** 1 Department of Psychology and Cognitive Science, University of Trento, 38068 Rovereto, Italy; alessandro.carollo@unitn.it (A.C.); andrea.bizzego@unitn.it (A.B.); 2 Department of Developmental Psychology and Socialization, University of Padova, 35131 Padova, Italy; paola.rigo.1@unipd.it; 3 Psychology Program, Nanyang Technological University, Singapore 639818, Singapore; albertlee@ntu.edu.sg (A.L.); psetoh@ntu.edu.sg (P.S.)

**Keywords:** in-group bias, multicultural context, system-justifying theories, functional magnetic resonance imaging

## Abstract

Recent migration and globalization trends have led to the emergence of ethnically, religiously, and linguistically diverse countries. Understanding the unfolding of social dynamics in multicultural contexts becomes a matter of common interest to promote national harmony and social cohesion among groups. The current functional magnetic resonance imaging (fMRI) study aimed to (*i*) explore the neural signature of the in-group bias in the multicultural context; and (*ii*) assess the relationship between the brain activity and people’s system-justifying ideologies. A sample of 43 (22 females) Chinese Singaporeans (*M* = 23.36; *SD* = 1.41) was recruited. All participants completed the Right Wing Authoritarianism Scale and Social Dominance Orientation Scale to assess their system-justifying ideologies. Subsequently, four types of visual stimuli were presented in an fMRI task: Chinese (in-group), Indian (typical out-group), Arabic (non-typical out-group), and Caucasian (non-typical out-group) faces. The right middle occipital gyrus and the right postcentral gyrus showed enhanced activity when participants were exposed to in-group (Chinese) rather than out-group (Arabic, Indian, and Caucasian) faces. Regions having a role in mentalization, empathetic resonance, and social cognition showed enhanced activity to Chinese (in-group) rather than Indian (typical out-group) faces. Similarly, regions typically involved in socioemotional and reward-related processing showed increased activation when participants were shown Chinese (in-group) rather than Arabic (non-typical out-group) faces. The neural activations in the right postcentral gyrus for in-group rather than out-group faces and in the right caudate in response to Chinese rather than Arabic faces were in a significant positive correlation with participants’ Right Wing Authoritarianism scores (*p* < 0.05). Furthermore, the activity in the right middle occipital gyrus for Chinese rather than out-group faces was in a significant negative correlation with participants’ Social Dominance Orientation scores (*p* < 0.05). Results are discussed by considering the typical role played by the activated brain regions in socioemotional processes as well as the role of familiarity to out-group faces.

## 1. Introduction

Over the past decades, recent urbanization and globalization trends have raised the rates of migration, especially towards well-established countries [1,2,3]. As a result of migration flows, many nations have become ethnically, religiously, and linguistically diverse. In other words, they have become multicultural countries [4,5]. In this context, a central challenge faced by modern multicultural societies is to understand the mechanisms underlying complex social interactions in order to promote social cohesion and harmony among cultural and ethnic groups [3,6,7,8].

People generally decode the complex social world through social categorization, i.e., perceiving themselves and others as members of social categories [9,10]. With reference to the social world, the ability to categorize and to generate inferences becomes functional for organizing and making sense of our knowledge about human features and complex social dynamics [10,11]. Social categorization processes are conducted effortlessly and in an almost totally spontaneous and continuous manner [10,12,13,14]. These processes are commonly observed in adults, but they emerge early in development and guide children’s social inferences and expectations [10,15,16]. After having assigned themselves and others into categories, people tend to show social preferences towards members of their own group (in-group) rather than members belonging to different groups (out-group). Scholars refer to this phenomenon as the “*in-group bias*”—that is, the tendency to favor in-group rather than out-group members [17]. At the perceptual level, people tend to show an advantage in recognizing emotions expressed by people that are part of their ethnic in-group [18,19]. An effect that in the literature has been renamed as “*ethnic bias*” or “*ethnic advantage*” [20]. Moral cognition and behaviors are also influenced by the in-group bias [21]. The roots of the in-group bias are documented already in infants and young children [21,22]. As argued by Dunham et al. [23], implicit in-group favoritism emerges quite rapidly and it remains relatively stable across developmental trajectories.

For the potentially negative consequences of the in-group bias in inter-group relationships, addressing ethnic diversity within a multicultural country becomes of social and political importance. Although social contact appears to be a factor that mitigates inter-groups conflicts [24], Putnam [25] noted that ethnic diversity in one’s proximal residential area is typically associated with lower levels of solidarity and trust among the neighborhood. Putnam describes this tendency to turn inwards with the term “hunkering down” [26]. Around the world, ethnic diversity is also oftentimes accompanied by acts of discrimination, violence, and marginalization [6,27].

Factors like people’s cognitive load, attentional capacity, and processing goals might all modulate the strength of the in-group bias and the subsequent activation and application of stereotyped knowledge [28,29,30,31,32]. Even task characteristics modulate social categorization [33]. Another factor that modulates the in-group bias is people’s beliefs. In particular, system-justifying theories consist of a set of ideals that legitimize and maintain a hierarchical social system, even when unequal, in order to preserve the status quo [34,35,36,37].

Within the first year of life, the initial familiarity preference is usually followed by selective processing and discrimination in terms of specific facial features. The own-race preference seems to result from exposure to the prototypical and homogeneous facial environment. From the literature, even short-term familiarization with people belonging to other racial groups appears to mitigate the own-race effect [38,39]. The researchers observed that the early deprivation of other-race faces in the first years of life interfered later on with adolescents’ emotion recognition skills towards out-group individuals and was associated with a heightened amygdala response towards out-group faces.

Cortical and subcortical brain regions show sensitivity to social categorization cues embedded in faces in a variety of experimental tasks based on abilities such as face perception, social categorization, and empathy [40,41,42,43]. On the one side, regions such as the amygdala and the fusiform gyrus may underlie the ability to detect and categorize other people by group membership, especially for face-based categorization tasks [42]. The fusiform gyrus, in particular, reportedly shows higher activation to in-group rather than out-group faces [44,45]. The amygdala, another key region underlying the in-group bias, shows more variability in terms of activation to group membership as compared to the fusiform gyrus [17]. The amygdala activation to racial out-group faces is also found to be correlated with implicit (but not explicit) measures of race attitudes [46]. Nevertheless, familiarity with out-group members seems to mitigate the amygdala activation to out-group individuals [46,47]. On the other side, more conscious and top-down monitoring and regulation strategies also have a role in modulating the magnitude of the in-group bias [17]. Such deliberate processes are associated with higher activity in the anterior cingulate cortex (ACC), especially in its dorsal part, and lateral frontal regions (e.g., dorsolateral prefrontal cortex (dlPFC)) [48,49,50]. Therefore, in the context of in-group bias, higher activity in the ACC might result from a perceived contrast between spontaneous in-group attitudes and cognitive intentions to act fairly and without bias [48,49]. As the ACC conflict signal increases, regions along the dlPFC get recruited to implement goal-directed and task-relevant behaviors [48,51,52,53]. The dlPFC implements control strategies not only over cognitive abilities but also over emotional responses. While higher activation in both the ACC and the dlPFC is related to more negative implicit attitudes towards the out-groups, only the activation in the dlPFC mediates the relation between implicit attitudes and poor performance in subsequent executive control tasks [48,53]. This highlights that the activity in the dlPFC is correlated with the successful implementation of cognitive control over negative attitudes towards out-group members. Enhanced neural activity to in-group rather than out-group members is also found in areas that are typically involved in socio-emotional processes. In particular, the higher activation in regions such as the medial prefrontal cortex (mPFC), the superior temporal sulcus (STS), the temporoparietal junction (TPJ), and the insula has been regarded as a marker of in-group favoritism [42,54,55,56,57,58].

The current fMRI study aimed to (*i*) explore the neural underpinnings of the in-group bias in a multicultural context; and (*ii*) assess the relationship between the activated brain regions and participants’ system-justifying ideologies. Based on the reviewed literature, we hypothesized enhanced activation in the fusiform gyrus and other brain regions involved in socioemotional processes (e.g., the mPFC, the STS, and the TPJ) to in-group (i.e., Chinese) rather than out-group (i.e., Indian, Arabic, Caucasian) face images. We also hypothesized that familiarity to out-group faces, specifically with typical out-group (i.e., Indian) faces, would mitigate these effects, especially in the activation of the fusiform gyrus. Finally, we hypothesized that higher scores in questionnaires assessing system-justifying ideologies would correlate with stronger activation in the areas underlying the in-group bias.

## 2. Materials and Methods

### 2.1. Participants

A sample of 43 Singaporean participants (n = 22 females) was recruited from the Nanyang Technological University (NTU) in Singapore. All participants had a Chinese ethnic background and they were aged 21 to 27 years old (*M* = 23.36; *SD* = 1.41). All participants were right-handed, showed normal or corrected-to-normal vision, and had no report of a history of psychological or neurological disorders. All female participants were not pregnant at the time of data collection. All participants were further asked not to consume alcohol, nicotine, and caffeine 24 h before their scan session. Furthermore, to avoid biases due to the exposure to other cultural and ethnic contexts, we ensured that all the recruited participants had not traveled outside Singapore for more than 2 months in the 6 months prior to data collection.

Ethical approval for the study was obtained by the NTU IRB (2017-01-029). All participants were informed of the voluntary nature of the study and they all provided written informed consent. Before their experimental session, all study participants were instructed about the study and they all took part in an MRI safety briefing. After each scan session, the aims of the study were made clear to participants and a monetary compensation of S$50 was provided.

### 2.2. Procedure

To address the aims of the study, the experimental paradigm was divided into two phases. The first one, in which participants completed two self-report questionnaires assessing their system-justifying ideologies in terms of Right-Wing Authoritarianism and Social Dominance Orientation. The second one, in which participants were enrolled in a paradigm of passive exposure to faces while in the fMRI scanner. The fMRI scanner was used to obtain both functional and anatomical brain scans. Face images were presented on a screen and participants viewed them from inside the fMRI scanner thanks to a mirror positioned at their eye level on the head coil. Participants were asked to simply view and pay attention to the displayed images. A question that checked participants’ attention appeared three times on the screen in non-predictable intervals.

### 2.3. Questionnaires

#### 2.3.1. Right Wing Authoritarianism Scale

The Right Wing Authoritarianism Scale was conceived by Altemeyer [59,60] to assess: (*i*) authoritarian submission; (*ii*) authoritarian aggression; and (*iii*) conventionalism [61]. These three domains would reflect the individual’s degree of submission to authority, aggression against those deviating from the norm, and maintenance of traditions, respectively [37]. The Right Wing Authoritarianism score derives from the responses to 32 self-report items. All items consist of a series of statements/opinions for which the participant has to indicate their degree of agreement. All items are rated along a 9-point Likert scale, ranging from −4 (“very strongly disagree”) to +4 (“very strongly agree”). The questionnaire shows high internal consistency, with a Cronbach’s alpha of 0.90 [62].

#### 2.3.2. Social Dominance Orientation Scale

Social dominance orientation is a concept derived from the social dominance theory and it reflects a person’s tendency to favor inequalities among social groups [63]. The Social Dominance Orientation Scale is a 14-item self-report questionnaire created by Pratto et al. [63]. All items consist of statements/opinions regarding group-based egalitarianism. Items are rated on a 7-point Likert scale, ranging from 1 (“strongly disagree”) to 7 (“strongly agree”). The final score corresponds to the mean response to the 14 items [64]. The questionnaire shows high internal reliability, with a computed Cronbach’s alpha of 0.83 [63]. In general, people reporting higher scores on the Social Dominance Orientation Scale tend to endorse discrimination in social hierarchies [37].

### 2.4. Visual Stimuli

All stimuli in the study consisted of images of ethnically-different faces.

Specifically, four types of face images were used and classified into in-group and out-group in regard to the participants’ ethnicity. Since all participants recruited in the study had a Singaporean Chinese ethnic background, in-group faces all consisted of images displaying faces with typical Chinese features. Conversely, out-group stimuli included three groups of faces: Indian, Arabic, and Caucasian faces. Out-group faces were further categorized as ethnically typical and non-typical in relation to the Singaporean context. Indian faces represented the typical out-group stimuli, while Arabic and Caucasian faces were considered non-typical out-group stimuli. To reiterate, typical faces for Singaporean Chinese participants consisted of Chinese (in-group) and Indian faces (out-group), while non-typical faces consisted of Arabic (out-group) and Caucasian (out-group) faces.

**Figure 1:**
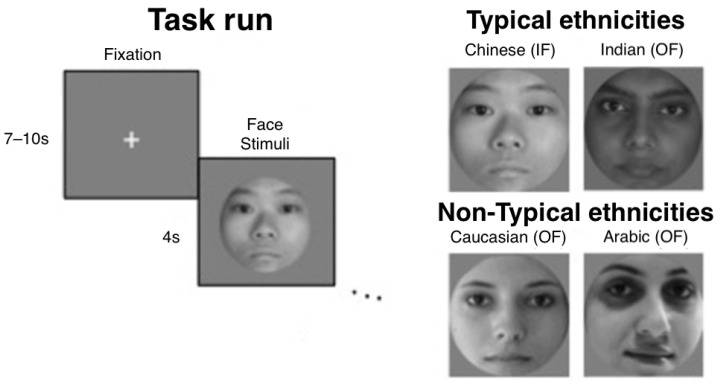
Experimental design in terms of task run (on the **left**) and presented stimuli (on the **right**).

All face images were presented in grayscale colors. All faces depicted females and were masked with a grey round window in order to make visible only the facial features (e.g., no hair or neck were visible). See Figure 1 for examples of adopted stimuli.

### 2.5. Experimental Task

To record the neural processing of in-group and out-group faces, participants took part in an fMRI paradigm. While in the fMRI scanner, participants were passively exposed to images of faces having typical ethnic features.

Each experimental trial began with a fixation cross presented for a randomized time interval ranging between seven to ten seconds. The fixation cross was followed by a face image presented for four seconds. The sequence of face image presentation was randomized for each participant. Overall, each participant took part in 32 experimental trials.

Figure 1 summarizes the experimental design of the current study.

### 2.6. MRI and fMRI Data Acquisition

A Siemens Magnetom Prisma 3-Tesla MRI Scanner with a 64-channel head coil was used to collect whole brain neuroimages. As an anatomical reference, a high-resolution T1-weighted MPRAGE sequence (192 slices; TR 2300 ms; TI 900 ms; flip angle 8 degrees; voxel size 1mm) was collected. Subsequently, functional images were collected adopting a gradient Echo Planar Imaging (EPI) sequence with 36 axial slices (271 slices; slick thickness 3mm with no inter-slice gap), and the following parameters: TR 2000 ms; TE 30 ms; flip angle 90 degrees; FOV 192 × 192 mm; voxel size 3 mm; interleaved. Head movements during the scan session were minimized by adopting external head restraints (i.e., neck padding).

### 2.7. fMRI Data Analysis

Data analysis was conducted adopting Statistical Parametric Mapping 12 (SPM12; http://www.fil.ion.ucl.ac.uk/spm/software/spm12, accessed on 11 April 2023) package for Matlab platform (version R2019a).

The pre-processing of functional images began by discarding the first two brain volumes of the functional time series. Images were then corrected for head movements and the mean of the realigned functional images was co-registered with the T1 anatomical brain image. Functional images were then normalized by following the Montreal Neurological Institute (MNI) stereotaxic standard space. Functional images were subsequently spatially (8-mm full-width half-maximum Gaussian kernel) and temporally (cut-off period 128 s) smoothed. For each participant, analytic design matrices were generated to model the onsets and duration of the face images displayed in each experimental trial as epochs convolved with a hemodynamic response function.

General Linear Models (GLMs) were performed both at the individual (1st-level) and at the group level (2nd-level) of analysis. GLMs were performed to assess the neural activation when perceiving ethnically in-group (i.e., Chinese) and out-group (i.e., Indian, Arabic, and Caucasian) faces based on the multicultural context of Singapore. For the 1st-level GLMs, a total of four different conditions were modeled as separate regressors. The four conditions consisted of displaying onsets of Chinese, Indian, Arabic, and Caucasian faces. Specific weight vectors were indicated to obtain contrast images. The contrasts of relevance were: *Chinese vs. Arabic + Indian + Caucasian faces*; *Chinese vs. Arabic faces*; *Chinese vs. Indian faces*; and *Chinese vs. Caucasian faces*. Together with the four conditions, six motion parameters were inserted as regressors in the 1st-level GLMs to control for head movements. The quality of all 1st-level contrast images was checked at *p* < 0.05 (uncorrected).

The 2nd-level analysis aimed at assessing for any group effect in the 1st-level contrast images and generating inferences across all participants for each contrast of interest. To do so, one sample *t*-test was computed for each contrast of interest. The test allows for retaining or rejecting the null hypothesis for which the aforementioned contrasts do not differ significantly from zero [65]. The significance threshold at the cluster-level for each 2nd-level one sample *t*-test was set at *p* < 0.0001 (uncorrected for multiple comparisons).

To assess the relationship between the activated brain regions and participants’ system-justifying ideologies, beta values were extracted from the active brain clusters and they were correlated with participants’ Right Wing Authoritarianism and Social Dominance Orientation scores. In particular, Pearson’s product-moment correlation test was adopted. Missing values in questionnaires total scores were replaced with the mean computed on the scores from the remaining participants. Specifically, two (n = 2) participants reported missing values on the Right Wing Authoritarianism Scale and their scores were replaced.

## 3. Results

### 3.1. In-Group Bias at the Neural Level

To assess the neural underpinnings of the in-group bias, the brain activity resulting from the exposure to in-group and out-group faces was compared. In particular, the neural activation for Chinese faces (in-group) was contrasted to the neural activation for Indian, Arabic, and Caucasian faces (out-group) altogether and, subsequently, in a pairwise way.

- *Chinese vs. Arabic + Indian + Caucasian faces*: higher activation in the right middle occipital gyrus [MNI coordinates (36, −70, 2)] and in the right postcentral gyrus [MNI coordinates (39, −31, 38)] was found. Conversely, no significant deactivation was found.
- *Chinese vs. Indian faces*: higher activation in the right postcentral gyrus [MNI coordinates (39, −31, 38)], in the right cuneus [MNI coordinates (30, −88, 23)], in the right middle occipital gyrus [MNI coordinates (39, −73, −1) and (54, −67, −13)], in the right middle temporal gyrus [MNI coordinates (45, −61, −4)], in the left middle temporal gyrus [MNI coordinates (−39, −58, 5)], and in the left middle occipital gyrus [MNI coordinates (39, −73, −1)] was found. Conversely, no significant deactivation was found.
- *Chinese vs. Arabic faces*: higher activation in the right supramarginal gyrus (at the border with the insula) [MNI coordinates (33, −25, 26)], in the left thalamus [MNI coordinates (−3, −7, −4)], in the left anterior cingulate [MNI coordinates (−3, 5, −7)], and in the right caudate [MNI coordinates (27, −37, 5)] was found. Conversely, no significant deactivation was detected.
- *Chinese vs. Caucasian faces*: no significant activation or deactivation was observed when comparing the neural activity for the exposure to Chinese and Caucasian faces.

All significant results are summarized in Table 1.

### 3.2. The Relationship between the Neural Representation of the In-Group Bias and System-Justifying Ideologies

**Figure 2:**
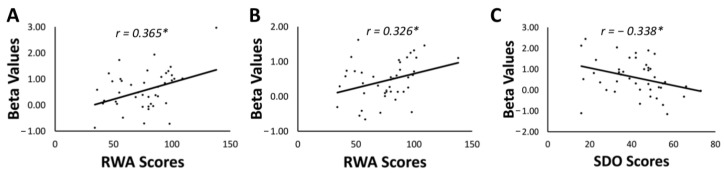
(**A**) Correlation between beta values in the right postcentral gyrus [MNI coordinates (39, −31, 38)] for the contrast Chinese > Arabic + Indian + Caucasian faces and participants’ scores in the Right Wing Authoritarianism (RWA) scale. (**B**) Correlation between beta values in the right caudate [MNI coordinates (27, −37, 5)] for the contrast Chinese > Arabic faces and participants’ RWA scores. (**C**) Correlation between beta values in the right middle occipital gyrus [MNI coordinates (36, −70, 2)] for the contrast Chinese > Arabic + Indian + Caucasian faces and participants’ scores in the Social Dominance Orientation (SDO) scale. * *p*< 0.05.

To assess the relationship between brain clusters activated in the aforementioned contrasts and participants’ system-justifying ideologies, beta values of brain activity were extracted and correlated with participants’ scores in the Right Wing Authoritarianism and Social Dominance Orientation questionnaires. Among all significant clusters of brain activity, two were activated in a significant positive correlation with the Right Wing Authoritarianism scores: the right postcentral gyrus [MNI coordinates (39, −31, 38)] when seeing Chinese rather than out-group (Arabic, Indian, and Caucasian) faces (*t*(41) = 2.51, *r* = 0.365, *p* = 0.01611, 95% CI [0.073, 0.600]; see Figure 2A), and the right caudate [MNI coordinates (27, −37, 5)] when seeing Chinese rather than Arabic faces (*t*(41) = 2.21, *r* = 0.326, *p* = 0.03269, 95% CI [0.029, 0.571]; see Figure 2B). Furthermore, the activity in the right middle occipital gyrus when participants were exposed to Chinese rather than out-group (Arabic, Indian, and Caucasian) faces was negatively correlated with the Social Dominance Orientation scores (*t*(41) = −2.302, *r* = −0.338, *p* = 0.02649, 95% CI [−0.580, −0.042]; see Figure 2C).

## 4. Discussion

The current work aimed to shed light on the inter-groups social dynamics in a multicultural country, such as Singapore. To do so, an fMRI study was conducted to explore the neural signature of the in-group bias in people living in Singapore and to assess the relationship between clusters of significant neural activation and participants’ self-reported system-justifying ideologies. To reflect the literature on the neural substrates of the in-group bias, we initially hypothesized that exposure to in-group (i.e., Chinese) rather than out-group (i.e., Indian, Arabic, Caucasian) face images would elicit enhanced neural activity in the fusiform gyrus and in other brain regions typically involved in socioemotional processes (e.g., the mPFC, the STS, and the TPJ). Moreover, in our hypothesis, familiarity and daily exposure to typical out-group (i.e., Indian) faces would mitigate the neural signature of the in-group bias, especially in regard to the fusiform gyrus activation. Ultimately, we hypothesized that higher scores in questionnaires assessing system-justifying ideologies would correlate with stronger activation in brain regions underlying the in-group bias.

Different patterns of brain activation in the contrasts of interests were observed in the current study.

Particularly, when participants were exposed to Chinese (in-group) rather than out-group (Arabian, Indian, and Caucasian) faces, brain activation was observed in the right middle occipital gyrus and in the right postcentral gyrus. In the literature, the middle occipital gyrus, besides being involved in the initial stages of face processing [66], relates to higher-level regions that control social cognition (e.g., superior temporal sulcus) and emotions processing [3,67,68,69]. In the current study, the activity in the right middle occipital gyrus for in-group rather than out-group faces resulted to be negatively related to participants’ SDO scores. Similarly to the middle occipital gyrus, the postcentral gyrus plays a role in mentalization, facial emotion recognition, and emotion processing with an embodied affective style [70,71,72,73,74,75]. The postcentral gyrus is also selectively activated when participants are asked to take the first-person perspective as compared to when they are asked to adopt a third-person perspective [74,76,77]. In the available literature, the role played by the postcentral gyrus in perspective-taking has been considered when explaining its preferential activation to in-group individuals. In fact, a selective response in the postcentral gyrus to cultural in-group rather than out-group members in a mental state decoding task has been documented by Adams Jr et al. [55]. As for the authors of the study, the finding suggests that people might adopt predominantly self-oriented simulations when decoding the mental state of another in-group member. In the current study, the preferential activity in the postcentral gyrus for in-group faces emerged to be positively correlated with the scores on the RWA.

When participants were exposed to Chinese (in-group) rather than Indian (typical out-group) faces, the right postcentral gyrus, the right cuneus, the bilateral middle occipital gyrus, and the bilateral middle temporal gyrus showed enhanced activation. In the literature, these areas appear to be involved in personal identity, social cognition, mentalization, and empathy. Similarly to the postcentral gyrus, the cuneus, together with the middle occipital and temporal gyri are all crucial in emotion processing and mentalization. In particular, the cuneus sustains emotion recognition and attribution, theory of mind abilities, and it shows higher activation when the individual’s attention is focused on face recognition [78,79]. Similarly to the middle occipital gyrus, the middle temporal gyrus is a central area in social cognition [80] and it is involved in social stimuli processing [81,82], face emotion expression processing, and in the understanding of others’ intentions [81,83]. In the literature, both the middle occipital and temporal gyri have been observed to be particularly active in response to in-group faces [69,84,85,86].

Furthermore, in the current work, when participants were exposed to Chinese (in-group) rather than Arabic (non-typical out-group) faces, a portion of the right supramarginal gyrus at the border with the insula, the left thalamus, the left anterior cingulate and the right caudate showed enhanced activation. Once again, as for the previous contrast, most of these brain regions are reportedly involved in socioemotional processes and empathy. In particular, the right supramarginal gyrus plays an important role in empathy as it subserves the distinction between self and other in the emotional context and it allows the person to overcome emotional egocentricity [87,88]. Like the postcentral gyrus, the supramarginal gyrus and the thalamus are among the main neural structures involved in the “mirroring” response, an automatic embodied simulation that facilitates emotional decoding of others [84,89]. Higher activity in the supramarginal gyrus to national in-group as compared to out-group members has been recently documented in the meta-analysis by Saarinen et al. [86]. The documented preferential activation to in-group members in brain areas typically involved in socioemotional processes finds agreement in the available literature [42,54,55,56,57,58,69,84,85,86]. However, it is worth noting that the current work did not adopt stimuli displaying emotional content, nor did it include an experimental paradigm to elicit empathy and mentalization, and, ultimately, it did not require participants to empathize with the observed faces. Therefore, although it might be hypothesized that the mere exposure to emotionally neutral in-group faces is enough to trigger the neural mechanisms of empathy and mentalization, the role of the activated brain regions and the reasons behind their activation still require clarification and future research.

Ultimately, in the current work, the caudate showed enhanced activity to Chinese (in-group) rather than Arabic (out-group) faces. The caudate is part of the striatum and, as such, it is a crucial structure for the anticipation of outcomes, instrumental learning, and reward processing [49,86,90]. In agreement with the current study, previous fMRI experiments have shown enhanced striatal activity when participants were shown images of in-group rather than out-group members [17,49,86,91]. In particular, the fMRI study by Beer et al. [91] documented enhanced caudate activity in response to racial in-group rather than out-group faces in a sample of white participants, together with a correlation between caudate activation and implicit preference for in-group members, assessed with the Implicit Association Task. Similarly, in the current study, not only the caudate showed preferential activity in regard to Chinese (in-group) rather than Arabic (out-group) faces. But, among all the clusters with significant brain activity, the caudate activation was the only one that resulted to be in a significant (positive) correlation with Right Wing Authoritarianism scores. In other words, the higher the authoritarian submission and adherence to social conventions, the higher the caudate activation as a function of in-group favoritism. In the literature, stronger Right Wing Authoritarianism scores have reportedly been related to slower response times for detecting out-group rather than in-group faces. It is therefore hypothesized that socio-political ideologies, such as Right Wing Authoritarianism might foster early racial bias through attentional disengagement towards out-group faces [92]. If so, for individuals reporting stronger Right Wing Authoritarianism ideologies, the correlated enhanced caudate activity might support selective attention to the valuable and rewarding in-group faces. Accordingly, the caudate seems specifically responsible for value-driven attentional orienting [93], and in particular, it is involved in the attentional capture by stimuli previously associated with social reward [94,95].

It is also worth discussing the effects that were initially expected to happen and eventually did not emerge in the results. First, in contrast to the previous results, no significant cluster of brain activation or deactivation emerged for *Chinese vs. Caucasian faces*. With regard to this, it might be beneficial to consider that all participants of the current study were recruited from the student population of the Nanyang Technological University in Singapore. In the Nanyang Technological University, a large part of the academic staff and student population has a Caucasian ethnic background. Thus, it is possible that the high exposure to Caucasian faces in the university environment might have had a role in mitigating the in-group neural signature, which is known to be reduced by familiarity and exposure to out-group members [17,46,47,96]. Familiarity and exposure to out-group members might also explain the fact that no activation was observed in brain areas typically recruited in face-based in-group favoritism, such as the fusiform gyrus. Once again, the existing literature suggests that the preferential neural activation to in-group faces in brain regions such as the fusiform gyrus reflects superior perceptual expertise and familiarization to in-group faces [17,96,97]. While we were expecting a mitigation of the in-group bias neural signature when comparing in-group (i.e., Chinese) to typical out-group (i.e., Indian) faces, such mitigation was not hypothesized for the contrasts in which in-group faces were compared to non-typical out-group (i.e., Arabic, Caucasian) faces. Once again, this non significant results might suggest that the daily exposure to typical and non-typical (but present in the context of Singapore) out-group members may completely mitigate the in-group neural signature. However, the current study did not include behavioral tasks or assessment of participants’ implicit tendencies towards in-groups and out-groups. Thus, the explanation of non-observed results through familiarity and daily exposure to out-groups requires great caution.

### Limitations of the Study

To interpret the observed and non-observed results of the current work, some limitations of the study must be carefully considered [3]. First, the sample size of the current work is relatively small. This might represent the main reason why results did not survive multiple comparison corrections. The initial trends in the results of this work should be tested by future studies including larger samples of participants. Furthermore, the study only assessed the neural substrates of the in-group bias in the ethnic group (i.e., Chinese) representing the majority in the Singaporean ethnic context. In fact, groups belonging to the majority and groups representing the minority do not perceive inter-group relations in the same way [98]. Thus, for a full understanding of the in-group bias and its neural substrates in a multicultural context, the same experiment should be repeated by involving participants from minority ethnic groups. Furthermore, the current study involved only young adult university students. This is a limitation, as age seems an important factor modulating the understanding of inter-group dynamics and social cognition [45,99]. In fact, age-related changes in the neural substrates of the in-group bias have been recently reported in the literature by Guassi Moreira et al. [45]. Another limitation of the study is that it only involved students at the Nanyang Technological University (Singapore). As the population of students in the Nanyang Technological University (Singapore) is strongly exposed to the Caucasian ethnicity, it cannot be considered representative of the daily context experienced by the general population in Singapore. An additional limitation of the study is that all stimuli adopted to elicit the in-group bias consisted of faces belonging to the female gender. In fact, some studies have shown that gender has an impact on the perception of the in-group racial bias [100]. Hence, future lines of research might try to extend the results of the current work by including stimuli portraying male faces. In this way, researchers would be able to control for any gender effect in the in-group favoritism and in its neural underpinnings [3]. Ultimately, the main limitation of the current work is that the experimental paradigm consisted of a passive exposure to visual stimuli. As the study did not include any behavioral task nor any implicit measures of attitudes towards in- and out-group members, no inference on participants’ mental attitudes towards in-group and out-group members can be made.

## Figures and Tables

**Table 1:** Regions of significant neural activation (*p* < 0.0001 (uncorrected)) for the following contrasts: *Chinese vs. Arabic + Indian + Caucasian faces*, *Chinese vs. Indian faces*, and *Chinese vs. Arabic faces*. No significant result emerged for the contrast *Chinese vs. Caucasian faces*.

Comparison of Interest	Brain Regions	BA	Left/Right	MNI Coordinates			Voxels at *p* (Uncorr.) < 0.0001	Mean t
				x	y	z		
Chinese > Arabic + Indian + Caucasian	Middle occipital gyrus		Right	36	−70	2	10	5.19
	Postcentral gyrus		Right	39	−31	38	20	4.85
Chinese > Indian	Postcentral gyrus		Right	39	−31	38	49	5.16
	Cuneus	19	Right	30	−88	23	26	4.91
	Middle occipital gyrus		Right	39	−73	−1	94	4.83
	Middle temporal gyrus		Right	45	−61	−4		4.62
	Middle occipital gyrus	37	Right	54	−67	−13		4.18
	Middle temporal gyrus		Left	−39	−58	5	10	4.39
	Middle occipital gyrus		Left	−39	−73	−1	18	4.37
Chinese > Arabic	Supramarginal gyrus (at the border with insula)	40	Right	33	−25	26	15	5.40
	Thalamus		Left	−3	−7	−4	20	5.06
	Anterior cingulate	25	Left	−3	5	−7		4.49
	Caudate	Caudate tail	Right	27	−37	5	17	4.97

## Data Availability

All the data are available at: https://doi.org/10.21979/N9/H0CPDV (accessed on 11 April 2023).
